# Prevalence, distribution on host’s body, and chemical control of camel ticks *Hyalomma dromedarii* in the United Arab Emirates

**DOI:** 10.14202/vetworld.2020.114-120

**Published:** 2020-01-16

**Authors:** Mohammad Ali Al-Deeb, Sabir Bin Muzaffar

**Affiliations:** Department of Biology, United Arab Emirates University, Al Ain, United Arab Emirates

**Keywords:** acaricide, *Hyalomma dromedarii*, prevalence, tick load, tick-borne disease, United Arab Emirates

## Abstract

**Background and Aim:**

Camel farming remains a part of the culture of the Arabian Peninsula although modern methods have greatly increased camel densities in the entire region. In the United Arab Emirates (UAE), camel production is threatened by tick parasitism. However, no study has considered assessing the magnitude of the problem in the UAE. We conducted a study evaluating tick richness, abundance, and spatial distribution of ticks on camels in farms near Al Ain, UAE. In addition, we conducted a survey of farm owners to determine the control methods used to eliminate camel ticks.

**Materials and Methods:**

Tick counts were made on 502 camels (*Camelus dromedarius*). For each examined animal, visual counts of ticks were made on the entire body segregating the counts by head, neck, forelegs, hump, abdomen, back legs, and tail area. In addition, a total of 70 camel owners from the study area were randomly selected and surveyed about the tick control methods.

**Results:**

*Hyalomma dromedarii* was the only species found during the study. The prevalence of ticks was 98% among the sampled animals. The mean intensity (tick load) was 25.8±2.4 ticks/host and the maximum number of ticks per animal was 102. Ticks were found in five vicinities that are on the border with Oman. The highest number of ticks on the body of the camel occurred on the tail area followed by the abdomen. Cypermethrin was the most commonly used acaricide (46.9%).

**Conclusion:**

The high abundance of ticks reported in this study calls for the establishment of a good management strategy. In addition, finding ticks in vicinities in the UAE that are on the border with Oman suggests a cross-border movement between the two countries. Therefore, studying this movement is important to understand its role in the global circulation of some *H. dromedarii* tick-borne diseases and the movement of acaricide resistance alleles among tick populations.

## Introduction

The Arabian Peninsula is a part of a hyper-arid region bounded by the Arabian Gulf on the northeast, the Gulf of Oman, the Indian Ocean on the southeast, and the Red Sea on the southwest. This region is of global geopolitical significance due to its vast oil and natural gas reserves [1]. The region has undergone rapid development, with close to 80 million people currently residing within the Arabian Peninsula. Camel farming has been of historical significance in the region and this has been greatly enhanced with development, with currently over 15 million camel heads in the Arabian Peninsula. The United Arab Emirates (UAE), in particular, has propelled into the forefront of development, resulting in the emergence of iconic cities such as Dubai and Abu Dhabi [1]. The tradition of camel farming has continued and currently, over 392,000 camel heads occur in the UAE constituting one of the highest densities of camels in the world [2]. Camel production remains an important component of the livestock industry, with a market for camel milk, meat as well as for the tradition of camel racing. Furthermore, with the establishment of Dubai as an important trade hub, livestock production has increased in general, with large numbers of livestock being imported from different countries in the region. This constitutes an important threat as vectors and associated pathogens could be imported into the UAE along with their hosts.

Camel production suffers from a number of threats including pathogenic viruses, bacteria, parasitic protozoans, helminths, and ticks, some of which could be imported from other countries through animal movements [3]. Camel ticks are important blood-feeding ectoparasites, which are able to transmit some viral and bacterial diseases to animals and people [4]. Acaricides are used extensively in livestock production systems in the UAE to control the number of ticks. Distribution of ticks, their biology, and host-parasite interactions have been poorly studied in the Middle East region. In adjacent Egypt, *Ornithodoros savignyi* [5], *Hyalomma Dromedarii*, and other *Hyalomma* spp. [6] are present; in Iran, several species were present including *Hyalomma anatolicum excavatum*, *Hyalomma* *marginatum marginatum*, *Hyalomma asiaticum asiaticum*, and *Rhipicephalus sanguineus* [7], while in Saudi Arabia, *O. savignyi* is present [8]. Williams *et al*. [9] conducted a tick survey in Oman on cattle and reported the presence of the following species: *Amblyomma variegatum, H. excavatum*, *H. dromedarii*, *H. anatolicum*, *Rhipicephalus pulchellus*, and *Rhipicephalus evertsi*. In addition, in Pakistan, several tick species were reported from livestock farms (*H. anatolicum*, *Rhipicephalus microplus*, *H. dromedarii*, and *Rhipicephalus turanicus*) [10]. *Hyalomma* ticks serve as vectors of theileriosis and rickettsiosis and are widespread in North Africa, Southern Europe, Middle East, Central Asia, and China [11-13]. In Sudan, several tick species were collected from camels including *H. dromedarii*, which comprised 72.22% of the total number of collected ticks [14]. The viral disease Crimean-Congo hemorrhagic fever (CCHF) is of great epidemiological importance to the region, being endemic to Iran [15,16] and widespread in Northern Africa [17]. The spatial distribution of *Hyalomma* ticks appears to overlap with CCHF distribution, thereby implicating ticks in this genus as an important vector [17]. The 1994-1995 CCHF outbreak in the UAE was of multisource origins possibly associated with the importation of CCHF virus-infected livestock and ticks [18]. *H. anatolicum*, a very common species in the region that feeds on domestic livestock, lizards, rodents, hedgehogs, hares, and humans is regarded as the major vector of CCHF, although both *Hyalomma impeltatum* and *Hyalomma truncatum* have also been implicated as vectors [17]. The kennel or brown dog tick, *R. sanguineus*, and the closely related *R. turanicus* that generally feeds on dogs can carry CCHF. A study in Oman [9] reported the presence of several tick species and concluded that the presence of clinical disease and the serological results for animals, humans, and infected *Hyalomma* ticks provides ample evidence of the presence of CCHF virus, suggesting that the virus could be more widespread than previously thought. Furthermore, Charrel *et al*. [8] reported one *O. savignyi* tick from Saudi Arabia contained Alkhurma hemorrhagic fever virus (AHFV) RNA, confirming for the 1^st^ time that it was a tick-borne flavivirus associating human AHFV cases with a history of tick bites. In addition, *Francisella*-like endosymbionts and *Rickettsia* species were found in *Hyalomma* species [19]. Roshdy [5] discovered a *Rickettsia*-like microorganism which was described, for the 1^st^ time, from the tick *O. savignyi* collected from camels in Egypt. Furthermore, another study confirmed the presence of *Rickettsia* in *H. dromedarii* in Egypt [20]. Loftis *et al*. [6] assessed the presence of rickettsial pathogens in ticks from domestic animals in Egypt. They reported the presence of several tick species in the genus *Hyalomma*, including *H*. *dromedarii*, and they detected *Anaplasma marginale*, *Coxiella burnetii*, and *Rickettsia aeschlim*, further highlighting the importance of *Hyalomma* ticks in the region. In another study, *C. burnetii* was detected in few *H. dromedarii* ticks in Egypt [21]. Razmi *et al*. [7] conducted a study to determine the population of ticks in infected cattle and to identify the tick vectors of bovine theileriosis (*Theileria annulata*) in an endemic area of Iran. The prevalence of ticks infesting cattle was 92.35% *H. excavatum*, 5.14% *H*. *marginatum*, 1.17% *H. asiaticum*, and 1.32% *R. sanguineus*. Moreover, *T. annulata* was reported in *H. dromedarii* in Egypt [22]. Chhabra and Khurana [23] mentioned that ectoparasites of camels and the injury and disease associated with them are more prevalent and more serious than is commonly realized. Infestations of *H. dromedarii* are characteristically heavy, causing widespread distress and morbidity in camels (possibly through their role as vectors of disease), thereby affecting the economy of camel rearing in diverse ways [3,23]. In addition, *R. aeschlimannii* was reported in *Hyalomma* spp. ticks from camels in Nigeria [24]. Thus, it is clear that ticks and tick-borne diseases are of great concern to the camel industry, but studies are limited and long-term studies exploring population dynamics or disease persistence are missing.

Several chemicals and methods are used to control ticks. El-Azazy [25] conducted a study using a pour-on application of the pyrethroid flumethrin as a control method of camel tick *H*. *dromedarii* in Saudi Arabia. No side effects of treatment were observed and his trial demonstrated that flumethrin is safe and effective when used to control ticks on camels. Furthermore, the pour-on method for insecticide application is fast and easy and is suitable for use by camel owners in the desert. El-Kelesh and El-Refaii [26] investigated the insecticidal effect of *Bacillus thuringiensis* var. *kurstaki* against *H. dromedarii* on experimentally infested rabbits in Egypt. They reported significant control effects. Chhabra and Khurana [23] mentioned that acaricidal control agents presently in use are not wholly satisfactory. There are several published records on the camel tick species of the Arabian Peninsula and neighboring countries; however, the information on ticks in the UAE is very limited.

The objectives of the current study were (1) to determine the species richness and abundance of ticks in camel farms in Al Ain, UAE, (2) to characterize the distribution of ticks on different microhabitats on the body of camels, and (3) to survey the methods of tick control used in the region.

## Materials and Methods

### Ethical approval

This study was carried out in strict accordance with the recommendations of the Animal Research Ethics Committee of the UAE University. The experimental protocol was approved by the UAE University Research Office.

### Study locations and tick visual counting

**Figure-1:**
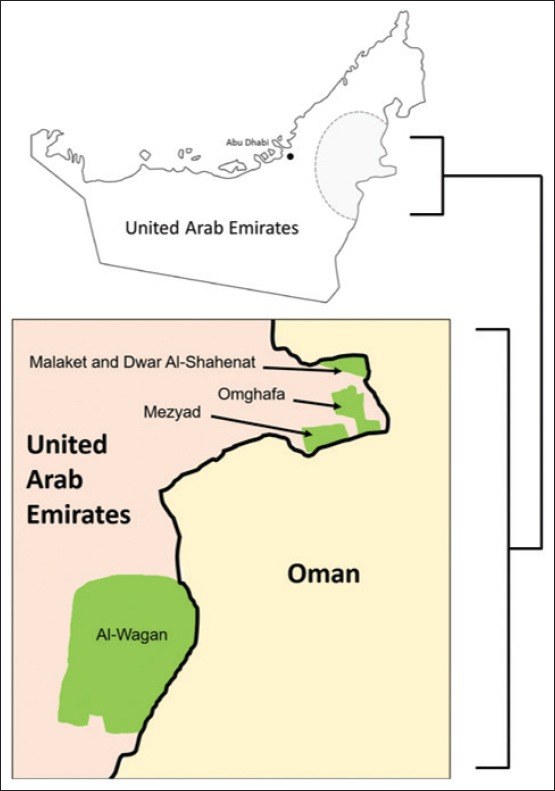
Upper map: The shaded area represents the locations of the camel farms visited in the current study. Lower map: Green areas are the possible points for cross-border tick movement between the United Arab Emirates and Oman [Source: Figure prepared by MAA].

Al Ain is a large city within the Abu Dhabi Emirate located approximately 120 km inland from either Dubai or Abu Dhabi cities. Twenty-one locations, which were visited during the sampling, represented all of the major camel breeding locations in the study area in Al Ain. Camels were reared on farms and housed in homestead, locally called (Al Izba), in which they were provided with feed and water. In addition, periodically, they were left to graze freely in the nearby desert where they enjoyed freedom and eating different plants. Tick counts were made on 502 camels (*Camelus dromedarius*) during spring and summer (March–August) in 2010 and 2011. For each examined animal, visual counts of ticks were made on the entire body segregating the counts by head, neck, forelegs, hump, abdomen, back legs, and tail area. For each animal, the collected ticks were placed in 50 ml plastic tubes and stored in −80°C freezer. In the laboratory, ticks were morphologically identified using a tick taxonomic key [27]. Furthermore, tick identification was confirmed by molecular techniques based on the cytochrome oxidase *c* subunit I gene [13]. All camels used in this study were intended for meat or milk production. The locations and numbers of the sampled animals are presented in Figure-1 and Table-1.

**Table-1:** Prevalence and load of *Hyalomma dromedarii* ticks on camels in Al Ain, United Arab Emirates.

Location	Number of camels	Tick load/camel			Tick prevalence (%)	Sampling date

		Mean±SE	Minimum	Maximum		
Malaket	12	36.8±1.0	32	44	100	June 2010
Dwar Al-Shahenat	8	37.5±2.6	28	48	100	
Al-Sad	12	36.2±2.2	22	48	100	
Maragh	18	33.9±2.3	18	52	100	
Mezyad	100	15.3±0.9	3	51	100	March 2011
Nahel	10	21.4±3.1	5	36	100	
Seeh Al-Salam	5	56.6±17.5	14	102	100	
Al-Nesoreya	7	7.3±2.9	0	22	71.40	
Al-Ajban	17	35.7±3.6	15	74	100	
Malaket	30	26.5±0.9	16	36	100	
Al-Dhahera	18	25±1.5	14	38	100	April 2011
Al-Arad	10	24.2±1.3	16	30	100	
Mezyad	19	19.6±1.6	10	36	100	
Omghafa	18	24±1.4	10	36	100	
Dwar Al-Shahenat	42	30.6±0.8	20	42	100	
Swehan	55	9.9±1.6	0	49	94.50	May 2011
Remah	5	16.8±1.0	14	20	100	
She’ab Al-Ghaf	56	25.8±1.0	12	44	100	
Maragh	14	24.3±1.1	18	30	100	
Al-Wagan	30	18.2±1.4	4	38	100	
Al-Selemat	16	17.1±1.5	10	30	100	June 2011
Total	502	25.8±2.4	0	102	98	

### Camel control survey

A total of 70 camel owners from the study area were randomly selected to answer two questions: (1) How do you control ticks on your camels and (2) if you use chemical control what chemical(s) do you use.

### Statistical analysis

The number of ticks in the following regions was quantified: head, neck, forelegs, hump, abdomen, back legs, and tail area. The distribution of ticks in each region had aggregated distributions. Thus, the mean intensity, mean abundance, and prevalence were all calculated for all regions [28]. Mean intensities and mean abundance values were compared between regions using bootstrap t-tests, and p-values were generated using 2000 replications. The prevalence of ticks was compared between regions using Fisher’s exact test and 95% confidence levels were calculated using the Clopper–Pearson method [28]. All comparisons were made using the Quantitative Parasitology Software Version 3.0 [28]. Responses of the questions on the tick chemical control were recorded in a Microsoft Excel sheet and the percentages were calculated.

## Results

**Figure-2:**
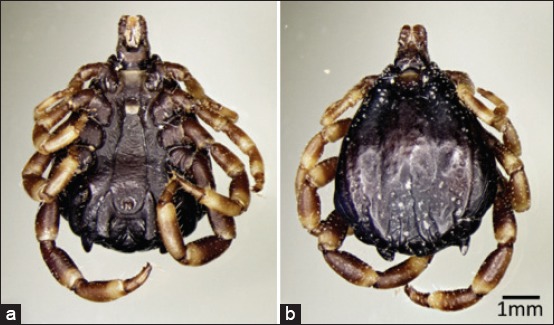
Camel tick *Hyalomma dromedarii* (*Acari*: *Ixodidae*) adult male: (a) Ventral and (b) dorsal.

All collected ticks were identified as *H. dromedarii* (Figure-2). The prevalence of *H. dromedarii* ticks was 100% in all sampled locations except in Swehan and Al-Nesoreya (94.5% and 71.4%, respectively), which made the overall prevalence to be 98% (Table-1). In general, the majority of camels were infested with 30-50 ticks; however, the maximum number of ticks per camel was recorded in Seeh Al-Salam (102) followed by Al-Ajban (74).

**Figure-3:**
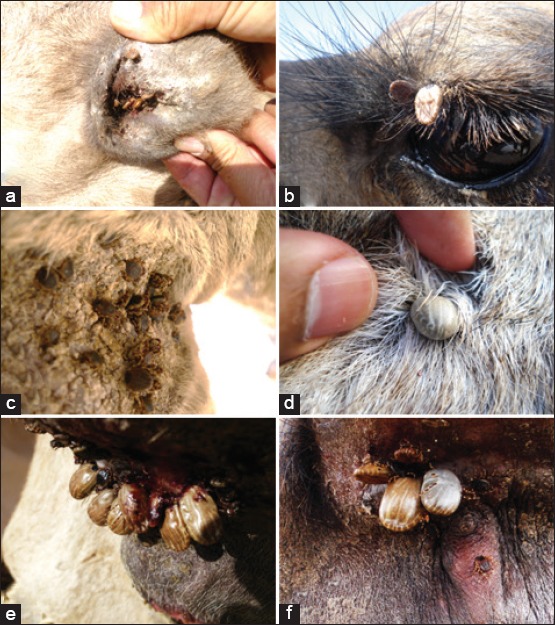
*Hyalomma dromedarii* ticks on different body parts of the camel: (a) Ear, (b) upper eyelid, (c) leg, (d) abdomen, and (e and f) tail area.

Ticks were found on different body parts (Figure-3). The body regions of camels differed in terms of prevalence, mean intensity, and mean abundance of ticks. The tail area had the highest prevalence (95%), mean intensity (6.25 ticks/infected host), and mean abundance of ticks (5.92 ticks/host, Table-2). The abdomen was the second most heavily infested region although this was not significantly lower than the tail (Table-2). All other regions were less infested with ticks in terms of prevalence, mean intensity, and mean abundance (all pairwise comparisons were not significant), with one exception. The forelegs had significantly higher tick prevalence, mean intensity, and mean abundance compared to the head (Table-2). In addition, the forelegs had higher prevalence and mean abundance (but not mean intensity) compared to the hump.

**Table-2:** Camel tick *Hyalomma* *dromedarii* prevalence, mean intensity, and mean abundance on examined animals.

Body region	Prevalence (95% confidence level)	Mean intensity (95% confidence level)	Mean abundance (95% confidence level)
Head	0.70 (0.66-0.74)^b^	2.82 (2.67-2.96)^b^	1.98 (1.82-2.13)^b^
Neck	0.64 (0.59-0.69)	3.05 (2.85-3.28)	1.97 (1.79-2.17)
Forelegs	0.79 (0.75-0.82)^b,c^	3.31 (3.06-3.73)^b^	2.61 (2.40-2.95)^b,c^
Hump	0.74 (0.70-0.78)	3.30 (3.06-3.58)	2.44 (2.22-2.68)
Abdomen	0.89 (0.86-0.92)^a,d^	4.48 (4.15-4.96)^a,d^	3.99 (3.66-4.38)^a,d^
Back legs	0.69 (0.65-0.74)	3.26 (3.08-3.43)	2.26 (2.09-2.45)
Tail	0.95 (0.92-0.96)^a,d^	6.25 (5.98-6.55)^a,d^	5.92 (5.61-6.23)^a,d^

a Significantly higher than head, neck, forelegs, hump, or back legs (p<0.001), in all pairwise comparisons),

b Forelegs significantly higher than head (p≤0.02) in all comparisons,

c Forelegs significantly higher than hump (p≤0.002) in pairwise comparisons,

d Tail significantly higher than abdomen (p≤0.002) in pairwise comparisons

**Figure-4:**
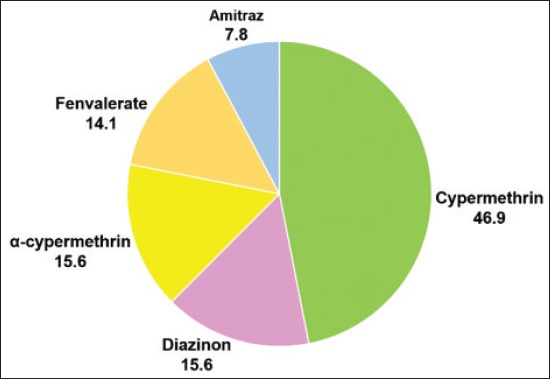
Pie chart showing the percentage of chemicals used in the control of the camel ticks *Hyalomma dromedarii*. Presented data are results of a survey of 70 camel owners in Al Ain, United Arab Emirates.

Concerning tick control, 69.6% of the camel owners indicated that they used a combination of manual tick removal and chemical control. However, 23.2% of the owners used chemical control alone and 7.2% removed the ticks manually. Cypermethrin was used by 46.9% of the camel owners on the infested animals followed by diazinon (15.6%), α-cypermethrin (15.6%), fenvalerate (14.1%), and amitraz (7.8%) (Figure-4).

## Discussion

Although *H. dromedarii* ticks are important blood-feeding ectoparasites, which can transmit diseases to camels and the people interacting with them, limited studies on tick-borne diseases have been done in the UAE. The current study provides needed information showing the magnitude of the tick load per animal and identifying tick-infested zones on the map of the study area. Therefore, this study will aid animal health authorities in the UAE to achieve more effective *H. dromedarii* control levels.

*H. dromedarii* ticks were detected almost on each sampled camel with very high prevalence. The high prevalence indicated that these ticks were endemic in all sampled camel farms in the Al Ain area. However, these high prevalence values could drop if a larger number (n>502) of camels were included in the study because there is more chance of sampling tick-free animals. We speculate that local farms provide a suitable environment, in which moisture and shelter from the harsh desert environment as well as suitable hiding locations are abundant. Thus, local farms could represent areas of refuge for ticks that could enhance their population size, which is otherwise not possible in the open desert environment. The high prevalence of ticks on camels indicates that a good tick management program is needed in the study area. Ticks feed on the blood of the infested animals and can transmit diseases among them. On average, the tick loads ranged between 7.1 and 56.6 ticks per animal, with loads as high as 102 ticks on one animal recorded in Seeh Al Salam. If such numbers of ticks are left unchecked, the infestation builds up overtime to levels that may negatively affect the host’s health. The present study agrees with other published studies in neighboring countries, which report the high prevalence of *Hyalomma* ticks. Razmi *et al*. [7] reported 92.35% prevalence of *H. excavatum* ticks infesting cattle and in addition, Abdullah *et al*. [20] reported that 91.9% of camels had been infested by *H. dromedarii*. The high prevalence of camel ticks presented in the current study must draw attention to the need for investigating the impact of these ectoparasites on camel and human health in the affected locations. We would like to emphasize the role of ticks in the transmission of diseases such as CCHF [18], theileriosis [11], and rickettsiosis [6]. In the UAE, a total of 625 *H. dromedarii* ticks, which were collected from the study area were screened for the presence of pathogens and some of them were positive for spotted fever group *Rickettsia* sp. and *T. annulata* [13]. Although, these two pathogens were found in low prevalence in the sampled tick population, their presence indicated that such pathogens were circulating among camels and the tick vectors. As a result, this can likely pose health risks to people living in rural areas and near camels.

In this study, finding *H. dromedarii* ticks in areas on the border with Oman such as Al-Wagan, Omghafa, Malaket, Mezyad, and Dawar Al-Shahenat sheds light on some of the probable dispersal mechanisms that give rise to tick infestations in both countries. One possible cross-border dispersal mechanism may occur when tick-infested camels graze in close proximity to the border fence and some ticks dislodge and disperse into Oman through contiguous habitat to infest animals on the opposite side of the fence. Another likely mechanism of tick dispersal may be when tick-infested alternative hosts (such as small rodents) move between both sides of the border fence. In addition, two-sided tick dispersal could play a major role in the introduction and the reintroduction of tick-borne diseases in the UAE and Oman. Moreover, the potential bilateral tick dispersal between the UAE and Oman could affect the gene flow in tick populations in both countries and this is a very important factor, especially for acaricide resistance development. Several studies documented the cross-border movement of different tick life stages, using various dispersal mechanisms, between neighboring countries [29-31] and this aspect of cross-border movements requires further study in the UAE.

Ticks were detected on different parts of the animal’s body. In some cases, they were found on the eyelids and inside the ears, although the largest number of ticks was recorded on the tail area. In this place, ticks find a good feeding niche, in which they hide under the tail and feed near the anal sphincter benefiting from the tender tissues and moisture. The abdomen was the second most heavily infested region. Overall, ticks made good use of every body part that could provide a suitable feeding surface and good shelter.

The majority of camel owners who had been surveyed in the current study combined manual tick removal and the use of chemicals to control ticks on their infested animals. It should be noted that manual tick removal is a very effective non-chemical that is an environmentally safe control option. However, it becomes labor intensive in large camel herds. In some of the visited camel farms, some chickens were observed feeding of the ticks from infested camels while they were sitting on the ground. Although, this “grooming” behavior could be considered as a natural biological control of ticks, it is not practical and poses a risk of infecting the chicken with some of the tick-borne diseases. The majority of camel owners used pyrethroid pesticides (cypermethrin, α-cypermethrin, and fenvalerate) in the chemical control of *H. dromedarii* ticks, while some of them used diazinon and amitraz. As with any use of chemical pesticide, there is always a chance for the development of resistance in the treated pest. In addition, chemical pesticides, especially systemic pesticides, can pose a human/animal health risk and an environmental hazard. The deployment of chemical acaricides in the field should be coupled with a resistance monitoring and management program. In addition, an integrated pest management system should be developed for *H. dromedarii* ticks in the UAE.

This study focused on the eastern part of the UAE. A survey of the entire country is in order. Future studies should focus on the geographic spread, distribution patterns, and loads of *H. dromedarii*, with special emphasis on cross-border movement to assess its impact on disease diversity and severity. In addition, future studies need to focus on identifying the factors affecting the high camel tick prevalence.

## Conclusion

This study revealed that *H. dromedarii* ticks had a high prevalence on the camels in Al Ain. Tick load was variable with very high loads on some animals, indicating that there is a need for a good tick management program. aThe occurrence of *H. dromedarii* ticks on camels in vicinities in the UAE that are on the border with Oman such as Al-Wagan, Omghafa, Malaket, Mezyad, and Dawar Al-Shahenat may mean a cross-border movement of the ticks between the two countries. Studying this movement should give a better understanding of its role in the global circulation of certain *H. dromedarii* tick-borne diseases and the movement of acaricide resistance alleles among tick populations. Although camel owners in the UAE used acaricides to combat with *H. dromedarii*, there is no study assessing the acaricide resistance status in the country. Therefore, future research should study resistance in tick populations and monitor its development.

## Authors’ Contributions

MAA designed and performed the study. MAA and SBM analyzed the data. MAA wrote the first draft of the manuscript. SBM participated in writing and editing the current version of the manuscript. MAA and SBM revised and approved the final manuscript.
